# Association between interleukin-6 gene polymorphism and iron regulation in hemodialysis patients infected with HCV

**DOI:** 10.1590/2175-8239-JBN-2019-0188

**Published:** 2020-07-27

**Authors:** Yasser B.M. Ali, Saad G. Moussa, Samar M. Shahen, Mohammed A. Dewir, Ibrahim H. El-Sayed

**Affiliations:** 1 University of Sadat City, Genetic Engineering and Biotechnology Research Institute (GEBR), Molecular Biology Department, Sadat City, Egypt.; 2 Desouk General Hospital, Hemodialysis Unit, Kafr El-Sheikh, Egypt.; 3 Kafr El-Sheikh University, Faulty of Science, Biochemistry Department, Kafr El-Sheikh, Egypt

**Keywords:** Hepcidins, Anemia, Renal Dialysis, Hepacivirus, Polymorphism, Genetic, Interleukin-6, Hepcidinas, Anemia, Diálise Renal, Hepacivirus, Polimorfismo Genético, Interleucina-6

## Abstract

**Backgrounds:**

Hepcidin is related to the pathogenesis of chronic renal failure anemia, which is considered a chronic inflammatory state as well as HCV infection. IL-6 stimulates the release of hepcidin from the liver, suppresses intestinal iron uptake, and releases iron from internal stores.

**Method:**

To detect the association between IL-6 gene polymorphism and anemia markers, 80 hemodialysis (HD) patients [40 negative HCV HD patients and 40 positive HCV HD patients] were studied by routine chemistry and complete blood count, in addition to the assessment of serum hepcidin, iron parameters [serum iron and serum ferritin], and hepatitis C markers. IL-6 polymorphism -174G/C was determined by MS-PCR, while IL-6 polymorphisms -597G/A and -572 G/C were detected by PCR-SSP.

**Results:**

Hepcidin was non-significantly elevated in HCV-positive compared with HCV-negative hemodialysis patients. A statistically significant difference was detected between the negative and positive HCV HD patients in frequencies of IL-6 -174 G/C and -597 G/A (P≤ 0.01 and P≤ 0.001, respectively). On the other hand, a non-significant difference was reported between negative and positive HCV HD patients in the frequencies of IL-6 -572 G/C.

**Conclusions:**

Our study indicated that IL-6 -174 G/C and -597 G/A polymorphisms may play a role in HCV susceptibility in HD patients. Additional prospective studies on a larger population are needed to confirm our findings.

## RESUMO

**Introdução:**

A hepcidina está associada à patogênese da anemia por insuficiência renal crônica, considerada um estado inflamatório crônico e também infecção por HCV. A IL-6 estimula a liberação de hepcidina a partir do fígado, suprime a captação intestinal de ferro e libera ferro das reservas internas.

**Método:**

Para detectar a associação entre o polimorfismo do gene IL-6 e os marcadores de anemia, 80 pacientes em hemodiálise (HD) [40 pacientes em HD, negativos para HCV; e 40 em HD, positivos para HCV] foram avaliados por exames químicos de rotina e hemograma completo, além da avaliação da hepcidina sérica, parâmetros do ferro [ferro sérico e ferritina sérica] e marcadores de hepatite C. O polimorfismo da IL-6 -174G/C foi determinado por MS-PCR, enquanto os polimorfismos de IL-6 -597G/A e -572 G/C foram detectados por PCR-SSP.

**Resultados:**

A hepcidina não esteve significativamente elevada em pacientes com HCV em comparação com pacientes em hemodiálise negativos para HCV. Uma diferença estatisticamente significativa foi detectada entre os pacientes em HD HCV negativos comparados aos positivos nas frequências de IL-6 -174 G/C e -597 G/A (P≤ 0,01 e P≤ 0,001, respectivamente). Por outro lado, foi relatada uma diferença não significativa entre pacientes em HD HCV negativos e positivos nas frequências de IL-6 -572 G/C.

**Conclusões:**

Nosso estudo indicou que os polimorfismos de IL-6 -174 G/C e -597 G/A podem desempenhar um papel na suscetibilidade ao HCV em pacientes em HD. Ainda necessitamos de estudos prospectivos adicionais em uma população maior para confirmar nossos achados.

## Introduction

Chronic renal failure is a slow, insidious, irreversible deterioration in renal function^1^. Prevalence is estimated to be 8-16% worldwide. Anemia is a common feature of chronic kidney disease (CKD) associated with poor outcomes. The current management of patients with anemia in CKD is controversial, with recent clinical trials demonstrating increased morbidity and mortality related to erythropoiesis-stimulating agents^2^. Patients on dialysis are frequently affected by multiple comorbidities that can directly or indirectly contribute to anemia. The systemic and chronic nature of these diseases leads to frequent inter-current events that can depress hemoglobin (Hb) levels^3^.

In hemodialysis (HD) patients, hepatitis C virus (HCV) infection is the most common cause of acute and chronic hepatitis, and it increases the risk for death^4^. In developed countries, the prevalence of anti-HCV seropositivity among patients on maintenance HD ranges between 5 and 60%^5^. The frequency of HCV is much higher in patients undergoing dialysis in less developed countries. In Egypt, the prevalence of HCV antibodies in hemodialysis patients ranges from 52.3 to 82.3%^6^. Patients with chronic HCV infection often have increased liver iron. However, little is known about the mechanism of iron accumulation in the liver^7^.

Hepcidin, a 25-amino acid peptide hormone, exclusively synthesized in the liver, is thought to be a key regulator of iron homeostasis. Hepcidin is induced by infection and inflammation^8^^-^ 10. Impaired hepcidin regulation may play a role in alterations of iron metabolism in HD patients with HCV infection^7^. Whereas both inflammation and iron loading induces hepcidin production, erythropoietic activity suppresses its production. In the case of inflammation, the primary mediator seems to increase interleukin-6 (IL-6) levels, which in turn cause the binding of signal transducer and activator of transcription 3 (STAT3) to the hepcidin promoter, increasing its activity^8^^,^ 11. IL-6, one of the well-studied pro-inflammatory cytokines, is a major mediator of the acute phase response^12^.

The number of single nucleotide polymorphisms (SNP) in the human genome is estimated to be about one million. As SNPs may alter gene expression and function, there is considerable interest in their inter-individual variability for susceptibility and disposition to diseases^13^. In HD patients, gene polymorphisms modulating IL-6 synthesis may represent a fair means for testing the link between IL-6 and the development of CKD^14^. In the present investigation, we analyzed IL-6 gene polymorphism in chronic HCV seropositive Egyptian patients on regular HD to evaluate the association between IL-6 and anemia markers.

## Materials And Methods

### Patients And Controls

This study was conducted on 80 unrelated Egyptian patients on regular chronic HD for at least one year. They were recruited from the hemodialysis unit in Desouk General Hospital, Kafr El-Sheikh, Egypt. All investigations were performed following the University of Sadat City, Health and Human Ethical Clearance Committee guidelines for Clinical Researches. The local ethics committee approved the study protocol, and oral informed consent was obtained from all patients.

HCV infection was diagnosed by the presence of HCV antibodies using enzyme-linked immunosorbent assay (ELISA) and confirmed by the presence of HCV-RNA using real-time PCR. For HBV infection, HBV surface antigen (HBsAg) was tested by ELISA. According to the presence of HCV infection, hemodialysis patients were divided into two groups. Group (1) included 40 hemodialysis patients with negative HCV infection. Group (2) included 40 hemodialysis patients with positive HCV infection. Both groups were negative for HBV infection.

Exclusion criteria were HD patients who were not compliant with hemodialysis for six months with more than three missed dialysis sessions/month, patients exposed to hospitalization, major surgeries, episodes of gastrointestinal tract bleeding, access clotting or bacteremia, or any other infections in 4 weeks before the blood draws, pregnancy, patients with polycystic kidney disease, HBV infection, hematopoietic disorders (including multiple myeloma), decompensated liver cirrhosis, hyperparathyroidism, patients treated with interferon and/or ribavirin, and/or patients with a history of blood transfusion in the last six months.

Blood samples were withdrawn, and the following biochemical parameters were measured: serum albumin level, serum total bilirubin level, serum alanine aminotransferase (ALT) level, serum alkaline phosphatase (ALP) level, serum aspartate aminotransferase (AST) level, urea, creatinine, sodium, and potassium. Complete blood count, total iron, serum ferritin levels, and erythropoietin were also estimated. The hepcidin level was measured by the quantitative sandwich enzyme immunoassay technique (Sun Red Company, China)

### Il-6 snps genotyping

blood samples were collected in EDTA sterile tubes. Genomic DNA was isolated from whole blood, according to the manufacturer’s instructions (Qiagen Ltd., UK).

IL-6 (-174 G/C) rs1800795 was genotyped using a mutagenic separated polymerase chain reaction (MS-PCR) method^15^. The reaction was done in one tube containing the two alleles. The PCR mixtures consisted of DreamTaq Green PCR Master Mix (2x) (Fermentas, Thermo Fisher Scientific Inc.), 10 pmol of each allele-specific primer, 10 pmol of antisense primer, and 100 ng of DNA. PCR cycling conditions consisted of 95°C for 10 min [1 cycle], followed by 94°C for 30 seconds, 66°C for 45 seconds, 72°C for 45 seconds [40 cycles], and finally 72°C for 7 min [1 cycle]. An amplicon of 136 bp (C allele) and 121 bp (G allele) was demonstrated using 4% agarose gel electrophoresis and the size of PCR products was determined relative to the migration of a 25 bp step ladder.

IL-6 -572 G/C rs1800796 and -597 G/A rs1800797 were genotyped using the sequence-specific primer method (PCR-SSP). The primers sequence for IL-6 -572 G/C and -597 G/A were designed using the tool at HTTP://www.ncbi.nlm.nih.gov/tools/primer-blast), according to Talaat et al. (Table 1). PCR reaction consisted of 25 µL in two tubes, one for each allele. Thermal cycling conditions were 94°C for 2 min (1 cycle), followed by 96°C for 25s, 70°C for 45s, and 72°C for 20s (5 cycles), followed by 96°C for 25 s, 65°C for 5s, and 72°C for 45s (11cycles), and finally 96°C for 25 s, 55°C for 60 s, and 72°C for 2 min (15 cycles). IL-6 -572 G/C primers resulted in an amplicon of 325 bp and 473 bp for IL-6 -597 G/A. The size of PCR products was determined relative to the migration of a 100 bp step ladder using 2% agarose gel electrophoresis.

**Table 1:** Primers used to detect polymorphisms of IL-6 in negative and positive HCV- HD patients.

Primer	Product size
**IL-6 (-174) G/C**	
Forward G: 5'-GCACTTTTCCCCCTAGTTGTGTCTTACG-3'	121 bp
Forward C: 5'GACGACCTAAGCTTTACTTTTCCCCCTAGTTGTGTCTTGAC-3'	136 bp
Reverse: 5'-ATAAATCTTTGTTGGAGGGTGAGG-3'	
**IL-6 (-572) G/C**	
Forward G: 5’-GGCCAGGCAGTTCTACAACAGCCG-3’	
Forward C: 5’-GGCCAGGCAGTTCTACAACAGCCC-3	325bp
Reverse: 5’-ATTAGTGACTCAGCACTTTGG-3’	
**IL-6 (-597) G/A**	
Forward G: 5’-AAGTAACTGCACGAAATTTGAGGG-3’	
Forward A: 5’-AAGTAACTGCACGAAATTTGAGGA-3’	473 bp
Reverse: 5’-TGTGCAATGTGACGTCCTTTA-3’	

### Statistical analysis

all statistical analyses were performed using the Statistical Package for Social Science (SPSS) version 19 (LEAD Technology Inc.). Data are presented as means with corresponding standard deviation (SD). Comparisons among different groups were performed by an independent*t*-test. Each polymorphism was examined in the control population to confirm that the distribution of the genotypes confirmed to Hardy-Weinberg expectations (http://ihg.gsf.de/cgi-bin/hw/hwa1.pl) (HWE). The online tool SNP stats (http://bioinfo.iconcologia.net/SNPstats) was used to perform the haplotype analyses and calculate the LD parameters (D′ and r^2^). The genotype, allele, and haplotype frequencies were compared between cases and controls using a chi-square test (*χ* 2). The odds ratio (OR) and 95% confidence intervals (CI) were calculated to assess the risk associated with a particular allele, genotype, or haplotype.

## Results

### Patient baseline characteristics

the baseline characteristics of the 80 chronic hemodialysis (HD) patients are given in Table 2. The sample included 49 males and 31 females with mean age of 52.26±12.28 years. The 40 negative HCV-HD patients were 27 males and 13 females with mean age 51.42±2.14 years, labeled as group 1 and the 40 positive HCV-HD patients were 22 males and 18 females with mean age 53.10±1.73 years, labeled as group 2. Iron level increased significantly (P≤0.001) in positive HCV-HD (391.65±32.43) in comparison with HCV-negative HD group (108.07±11.57) (Table 2). Similarly, Hb, hematocrit, and ferritin (9.81±0.24, 28.54±0.78, 998.62±90.19, respectively, were significantly elevated (P≤0.001, P≤0.001, P≤0.01, respectively) in HCV-positive HD patients compared with HCV-negative HD patients (8.36±0.29, 25.61±1.31, 276.77±53.66, respectively). No statistical difference was found in hepcidin level in HCV-positive HD patients (595.86±52.96) compared to HCV-negative HD patients (570.64±59.38).

**Table 2:** Demographic data and markers of iron status of the study population including.

	HCV –ve HD	HCV + ve HD	P
	N= 40	N= 40	
Demographic data			
Gender (Male / Female)	27/13	22/18	NS
Age (years)	51.42 ± 2.14	53.10 ± 1.73	NS
HD duration (years)	2.20 ± 0.41	4.90 ± 0.63	P < 0.001
Iron parameters			
Hemoglobin (g/dL)	8.36 ± 0.29	9.81 ± 0.24	P < 0.001
Hematocrit (%)	25.61 ± 1.31	28.54 ± 0.78	P < 0.001
MCV (fl)	83.94 ± 1.09	87.08 ± 1.15	P < 0.001
MCH (pg)	28.38 ± 0.44	29.96 ± 0.45	P < 0.01
MCHC (g/dL)	33.91 ± 0.30	41.98 ± 7.50	NS
Ferritin (ng/mL)	276.77 ± 53.66	998.62 ± 90.19	P < 0.01
Iron (µg/dL)	108.07 ± 11.57	391.65 ± 32.43	P < 0.001
Hepcidin (ng/mL)	570.64 ± 59.38	595.86 ± 52.96	NS
Erythropoietin dose (IU/week)	4480 ± 200	4800 ± 240	NS

HCV-negative and positive HD patients. All data are presented as mean ± SD. N: number of cases, MCV: mean corpuscular volume, MCH: mean corpuscular hemoglobin, MCHC: mean corpuscular hemoglobin concentration, RBCs: red blood cells count and NS: non-significant. Differences between the 2 groups were compared by independent t-test. P < 0.05 is considered significant.

### Il-6 genotypes and allele frequencies

the frequency of the -572 G/C genotypes for IL-6 did not deviate significantly from the HWE equilibrium. However, the values predicted by the assumption of the HWE were different to those observed for Il-6 -174 G/C genotype GG, GC, and CC [9 (22.5%), 31 (77.5%), 0 (observed) vs. 15.01 (37.5 %), 18.99 (47.5%), 6.01 (15%) (predicted) (P≤0.001)] in HCV-negative HD patients. The frequency of IL-6 -597G/A genotypes significantly deviated from the HWE for GG, GA, AA [8 (20%), 29 (72.5%), 1 (2.5%) (observed) vs. 13.68, 17.64, 5.68 (predicted) (P≤0.001)] in HCV-positive HD patients.

The distribution of IL-6 alleles and genotypes are shown in Table 3. A statistically significant difference was detected between the HCV negative and positive HD patients in frequencies of IL-6 -174 G/C (P≤0.01). There was a significant increase in the frequency of -174 GG genotype (P<0.01, OR=4.66, 95%CI=1.76-12.31) in HCV-positive HD patients compare to HCV-negative HD patients and a significant decrease in the frequency of -174 GC genotype (P<0.01, OR=0.16, 95%CI=0.06-0.45) in HCV-positive HD patients compare to negative. The frequencies of G alleles were also higher among HCV-positive HD patients than negative (77.5% vs. 61.25%, P≤0.05, OR=2.17, 95%CI=1.09-4.35). On the other hand, no significant difference in the SNP frequency at the IL-6 -572 was recorded between HCV-positive and -negative HD patients

**Table 3:** Distributions of IL-6 (-174 G/C, - 572G/C, and – 597G/A) genotypes and allele frequencies in HCV-Negative and HCV-Positive HD patients.

IL – 6 variants	HCV –ve HD N= 40	HCV +ve HD N= 40	P Value	OR (95% CI)
**IL-6 (-174G/C) Genotype**				
G/G				
G/C	9 (22.5%)	23(57.5%)	P<0.01	4.66(1.76-12.31)
C/C	31(77.5%)	16(40%)	P<0.01	0.16(0.06-0.45)
GCCC	0 (0%)	1(2.5%)	NS	1.02 (0.97-1.07)
GCCC	31(77.5%)	17(42.5%)	P<0.01	0.21(0.08-0.56)
IL-6 (-174G/C) Allele Frequency				
G	49 (61.25%)	62(77.5%)	P<0.05	2.17(1.09-4.35)
C	31(38.75%)	18(22.5%)	P<0.05	0.45 (0.23-0.91)
**IL-6 (-572 G/C) Genotype**				
G/G	29 (72.5%)	34(85.0%)	NS	2.14(0.70-6.53)
G/C	8 (20.0%)	5(12.5%)	NS	0.57(0.16-1.92)
C/C	1(2.5%)	0	NS	0.97(0.92-1.02)
GCCC	9(22.5%)	5(12.5%)	NS	0.49(0.14-1.62)
**IL-6 (-572 G/C) Allele Frequency**				
G	68(87.2%)	73(93.6%)	NS	2.14(.69-6.60)
C	10(12.8%)	5(6.4%)	NS	0.46(0.15-1.43)
**IL-6 (-597 G/A) Genotype**				
G/G	27(67.5%)	8(20.0%)	P<0.001	0.12(0.04-0.33)
G/A	12(30.0%)	29(72.5%)	P<0.001	6.15(2.33-16.21)
A/A	0	0		
GAAA	12(30.0%)	29 (72.5%)	P<0.001	6.15(2.33-16.21)
**IL-6 (-597 G/A) Allele Frequency**				
G	66(84.6%)	45(60.8%)	P<0.01	0.28(0.13-0.61)
A	12(15.4%)	29(39.2%)	P<0.01	3.54(1.63-7.67)

OR, odds ratio; 95% CI, 95% confidence interval. NS, non-significant. The genotype, allele and frequencies were compared using a chi-square test (χ2). P < 0.05 is considered significant.

Analysis of IL-6 -597 G/A SNP revealed a significant difference in the distribution of different genotypes between HCV-positive and -negative HD patients (P≤0.001). HCV-positive HD patients had increased frequency of IL-6 -597 G/A vs. HCV-negative HD patients (P≤0.01, OR=6.15, 95%CI=2.33-16.21). The frequencies of A alleles were also higher among HCV-positive HD patients compared to negative (39.2% vs. 15.4%, P≤ 0.01, OR=3.54, 95%CI=1.63-7.67).

We analyzed the linkage disequilibrium (LD) pattern between the SNPs. There was LD between IL-6 -174 G/C and IL-6 -572 G/C (r^2^= 0.00086 and D’ 0.9975), between IL-6 -174 G/C and IL-6 -597 G/A (r^2^= 0.0038 and D’= 0.7486), and between IL-6 -572 G/C and IL-6 -597 G/A (r^2^= 0.00068 and D’= 0.3699).

As shown in Table 4, the haplotype frequencies of IL-6 -174 G/C, -572 G/C, and -597G/A in HCV-positive HD patients demonstrated a significant increase in the IL-6 GGA and CGA haplotypes (P≤0.01 and P≤0.001 respectively). At the same time, there was a significant reduction in the CGG haplotype (P≤0.01).

**Table 4:** IL-6 (-174G/C, -572G/C, and-597G/A) haplotypes in HD patients.

IL – 6 variants	HCV –ve HD N= 40	HCV +ve HD N= 40	P Value	OR (95% CI)
GGG	37.8%	52.52%	-	1.00
CGG	39.02%	9.28%	P<0. 01	0.09 (0.02 - 0.36)
GGA	9.49%	20.81%	P<0.01	8.71 (2.09 - 36.26)
GCA	6.69%	4.87%	NS	2.08 (0.14 - 31.21)
GCG	7%	0	NS	0.00 (-∞-∞)
CGA	0	12.09%	P<0.001	0.00 (-∞-∞)

OR, odds ratio; 95%CI, 95% confidence interval. NS, non-significant. The genotype, allele, and frequencies were compared using a chi-square test (χ^2^). P < 0.05 is considered significant.

### Association between markers of anemia and il-6 gene polymorphisms in hcv-hd patients

In HCV-negative HD patients, anemia markers did not significantly differ in the genotypes IL-6 -572 G/C and IL-6 -597. Hepcidin level increased significantly (P≤0.05) in IL-6 GC genotype (640.18± 399.44) in comparison to IL-6 GG genotype (331.09±78.14). In contrast , ferritin level was significantly elevated (P≤0.01) in IL-6 GG genotype (307.54 ±372.98) than IL-6 GC genotype ( 167.34 ±93.34) (Table 5). Anemia markers did not differ significantly in any of the studied IL-6 SNPs in HCV-positive HD patients (Table 6).

**Table 5:** Markers of iron status of IL-6 (-174 G/C, -572 G/C and -597 G/A) genotypes in HCV-negative HD patients.

	Hemoglobin	Hematocrit	RBCs	MCV	MCH	MCHC	Ferritin	Iron	Hepcidin
**IL-6 (-174)**									
GG	9.41± 1.56	27.22± 3.98	3.28±0.43	83.27± 6.63	28.62±2.64	34.42± 1.86	896.06 ±628.30	295.22±106.17	331.09±78.14
GC	8.05± 1.84	25.14 ±9.20	4.58 ±6.84	84.14 ±7.07	28.31 ±2.91	33.76 ±1.98	1028.39 ±560.03	419.64±219.34	640.18± 399.44
P value	NS	NS	NS	NS	NS	NS	NS	NS	P<0.05
**IL-6 (-572 G/C)**									
GG	8.16 ±1.81	25.28± 9.02	3.72 ±4.93	84.40 ±6.59	28.52± 2.57	33.91 ±1.80	1075.51± 546.50	422.77± 199.54	590.86 ±385.79
GC	8.74 ±1.82	26.06 ±5.49	6.53 ±9.56	82.55± 8.68	27.73 ±3.94	33.59± 2.53	633.99± 650.37	295.88± 208.48	516.53 ±370.94
CC	11.7	32.1	3.96	81.1	29.5	36.4	1532.21	193	376.74
P value	NS	NS	NS	NS	NS	NS	NS	NS	NS
**IL-6 (-597 G/A)**									
GG	8.35± 1.97	24.53 ±5.47	4.88 ±7.16	83.41 ±7.33	28.24± 3.03	33.95 ±2.10	1015.30± 516.62	403.57± 192.25	566.82 ±363.55
GA	8.39± 1.63	28.13± 12.73	2.93 ±0.58	85.20± 5.88	28.73±2.37	33.82 ±1.66	959.71± 704.35	363.83±239.40	579.57 ±419.08
**AA**	-	-	-	-	-	-	-	-	-
P value	NS	NS	NS	NS	NS	NS	NS	NS	NS

All data are presented as mean ± SD. N: number of cases, RBCs: Red blood cells count, MCV: Mean corpuscular volume, MCH: Mean corpuscular hemoglobin, MCHC: Mean corpuscular hemoglobin concentration, and NS: non-significant. Differences among the groups were compared by ANOVA. P < 0.05 is considered significant.

**Table 6:** Markers of iron status of IL-6 (-174G/C, -572 G/C and -597 G/A) genotypes in HCV-positive HD patients.

	Hemoglobin	Hematocrit	RBCs	MCV	MCH	MCHC	Ferritin	Iron	Hepcidin
**IL-6 (-174 G/C)**									
GG	9.97± 1.56	28.86± 4.81	3.37±0.63	86.32± 7.50	29.86±3.20	47.68± 2.51	307.54 ±372.98	112.04±83.11	605.19±347.86
GC	9.68± 1.55	28.05 ±5.42	3.21 ±0.58	87.78 ±7.19	30 .29±2.37	34.61 ±1.23	167.34 ±93.34	99.93±60.01	600.88± 329.07
CC	8.40	29.00	3.10	93.70	27.00	28.90	1320.00	147.00	300.83
P value	NS	NS	NS	NS	NS	NS	P<0.01	NS	NS
**IL-6 (-572 G/C)**									
GG	9.74 ±1.46	28.29±4.44	3.27 ±0.58	87.09 ±7.35	29.97± 3.00	43.04 ±50.70	292.61± 357.69	114.45± 75.80	594.41 ±329.20
GC	10.34 ±2.09	30.28 ±8.17	3.46 ±0.71	87.04± 7.69	29.90 ±1.80	34.54± 2.30	165.90± 134.61	63.40± 23.29	606.01 ±415.49
CC	-	-	-	-	-	-	-	-	-
P value	NS	NS	NS	NS	NS	NS	NS	NS	NS
**IL-6 (-597 G/A)**									
GG	10.13± 0.86	30.09 ±2.56	3.53 ±0.45	85.94 ±7.28	28.93± 3.21	60.99 ±90.66	397.32± 452.57	140.63± 76.54	566.54 ±315.84
GA	9.69± 1.72	27.95± 5.52	3.20 ±0.62	87.52± 7.38	30.35±2.67	34.77 ±1.66	231.04± 281.83	95.72±69.24	606.98 ±346.68
AA	-	-	-	-	-	-	-	-	-
P value	NS	NS	NS	NS	NS	NS	NS	NS	NS

All data are presented as mean ± SD. N: number of cases, MCV: Mean corpuscular volume, MCH: Mean corpuscular hemoglobin, MCHC: Mean corpuscular hemoglobin concentration, RBCs: Red blood cells count and NS: non-significant. Differences among groups were compared by ANOVA. P < 0.05 is considered significant.

## Discussion

High circulating levels of IL-6 have been documented in several clinical inflammatory conditions including liver diseases such as viral chronic hepatitis^16^, and various diseases in patients with end-stage renal disease^17^^,^ 18.

Studies on the cytokine gene polymorphisms suggest that inheritance of some genotypes related to polymorphisms of cytokine genes, such as the IL-6 gene, which clearly affect cytokine production and maybe host genetic factors associated with the progression of HCV^19^. With this overview, the present study was planned to identify the relationship between IL-6 and iron regulation in chronic hepatitis C seropositive Egyptian patients on regular HD.

HD duration was significantly longer in HCV-positive HD patients than in HCV-negative HD patients, as patients with HCV infection spent a significantly longer time (P≤0.001) on hemodialysis than those without HCV infection. These findings were supported by Saifan et al.^20^.

No significant difference in the hepcidin level was found between groups. This result is in accordance with the data of Fujita et al.^21^ who stated that there is no relation between HCV RNA load and serum hepcidin and supported by data of Ibrahim et al.^22^ who concluded that there is no significant difference in hepcidin level between HCV-negative and -positive HD patients.

This may be explained by a multiplicity of factors that influence hepcidin level. While inflammation, as well as HD treatment may increase its level, both anemia and iron deficiency may decrease its level. Moreover, infection with HCV may impair liver ability to secrete hepcidin, which might have important implications in the treatment of anemia in HD patients infected with HCV^22^.

There was a highly significant (P≤0.001) elevation of iron level among HCV-positive HD patients. At the same time, ferritin was also highly elevated (P≤0.01) in HCV-positive than -negative HD patients. These results agree with the study of Shan et al.^23^ in the US population, who stated that there is an association between HCV infection and higher levels of serum iron and ferritin. Besides, Sabry et al.^24^ stated that patients with end-stage renal disease and HCV-positive appear to have higher serum iron and TS compared to HCV-negative patients; moreover, there was a significant difference in serum iron and ferritin between groups.

Our study showed an increase in the frequency of IL-6 -174 G/C G/G, C/G, and GCCC genotypes in the HCV-positive HD patients than -negative, suggesting that susceptibility to HCV-positive HD patients may influence the frequency of IL-6. Concerning allele frequency, a non-significant change was demonstrated in G and C alleles in both groups, although the G allele was severely decreased compared to the C allele. These results are in agreement with Bennermo et al.^25^, who concluded that IL-6 gene was associated with a variety of major diseases as HCV. Also, our results match with Nasr et al.^26^, who observed that IL-6 genotype and polymorphism was related to the presence and outcome of HCV. While Giannitrapani et al.^27^ found no significant difference regarding neither genotype nor allelic frequencies of the polymorphism studied among their three groups. Moreover, Giannitrapani et al.^28^ proved that there is the possibility of a genetic association between IL-6 -174 G/C polymorphism and some specific liver diseases as they observed a correlation between the presence of the high producer genotype (GG) and a worse evolution of the HCV.

The polymorphism at position IL-6 -174 G/C is one of several IL-6 polymorphisms that have been suggested to affect the IL-6 expression^29^^,^ 30 According to our findings, there is evidence for a genetic association between the genotype of IL-6 -174 G/C except C/C, and the susceptibility to the HCV-positive HD patients was demonstrated. This result is contradictory to the results of Hirschhorn et al.^31^ and Rothman et al.^32^. The study of Liu et al.^33^ showed null associations between IL-6 -174 G/C and several common types of cancer, including breast, colorectal, prostate, lung, gastric cancer, lymphoma, multiple myeloma, and melanoma.

When studying IL-6 polymorphism at position -597 G/A there was a highly significant difference observed in carriage rate of ‘A’ allele in HCV-positive HD patients and highly significant in ‘G’ allele in HCV-negative HD patients (P<0.01) for both groups. These results agree with Park et al.^34^ who studied the association of IL-6 among the Korean population with hepatitis and Falleti et al.^35^ who observed that IL-6 -597 G/A polymorphism was related to the presence and outcome of HCV infection. Also, Cussigh et al.^36^, who reported the IL-6 -597 G/A, appear to favor a progressive HCV disease. In contrast to our study, Lu et al.^37^ found no significant difference in the IL-6 -597 G/A allele or genotype frequencies between the HCV patients and the control group.

Pro-inflammatory cytokines interact with hematopoiesis at various stages. Synthesis of hepcidin and ferritin are induced by IL-6^38^. Genetic polymorphisms of proinflammatory cytokine genes such as IL-6 might play a crucial role in anemia caused by chronic renal diseases^39^.

There was a significant correlation (P<0.05) between IL-6 -174 G/C GC and hepcidin levels in the HCV-negative HD population. In HD patients, the higher production for IL-6 has been associated with IL-6 -174 G/C GC genotype^40^. Thus, hepcidin level may be increased in IL-6 (-174 G/C) GC genotype patients, and this supports our result. On the other hand, ferritin level showed a significant correlation (P<0.01) with IL-6 (-174 G/C) CC genotype. Sabry et al.^24^ proved that serum ferritin was significantly increased in HCV-positive HD patients than in HCV-negative HD patients. Genetic polymorphism of IL-6 did not show any correlation with anemia in HCV-positive HD patients.

## Conclusions

In conclusion, our preliminary study indicated that IL-6 (-174 G/C and -597 G/A) polymorphisms may play a role in HCV susceptibility in HD patients, and IL-6 -174 G/C polymorphisms may play a role in the regulation of hepcidin and iron levels in HD patients. Finally, more extensive prospective studies are needed to confirm our findings.

## References

[1] Becherucci F, Roperto RM, Materassi M, Romagnani P (2016). Chronic kidney disease in children. Clinical Kidney Journal.

[2] Jha V, Garcia-Garcia G, Iseki K, Li Z, Naicker S, Plattner B, Saran R, Wang AY, Yang CW (2013). Chronic kidney disease: global dimension and perspectives. The Lancet.

[3] Brattich M (2007). Comorbid diseases in patients on dialysis: the impact on anemia. Nephrology Nursing Journal.

[4] Agarwal SK, Dash SC, Gupta S, Pandey RM (2009). Hepatitis C virus infection in haemodialysis: the 'no-isolation'policy should not be generalized. Nephron Clinical Practice.

[5] Etik DO, Ocal S, Boyacioglu AS (2015). Hepatitis C infection in hemodialysis patients: A review. World journal of hepatology.

[6] Soliman AR, Momtaz Abd Elaziz M (2012). Evaluation of an isolation program of hepatitis C virus infected hemodialysis patients in some hemodialysis centers in egypt. ISRN nephrology.

[7] El Said HW, Abou Seif KH, Ahmed YS, Abou Elleil HA, El Said TW, Behairy MA, Mohamed MM, Ahmed FA (2018). Relationship of serum haemojuvelin and hepcidin levels with iron level and erythropoietin requirement in prevalent hepatitis C virus positive haemodialysis patients. Nephrology.

[8] Nemeth E, Rivera S, Gabayan V, Keller C, Taudorf S, Pedersen BK, Ganz T (2004). IL-6 mediates hypoferremia of inflammation by inducing the synthesis of the iron regulatory hormone hepcidin. The Journal of clinical investigation.

[9] De Domenico I, Ward DM, Kaplan J (2008). Regulation of iron acquisition and storage: consequences for iron-linked disorders. Nature reviews Molecular cell biology.

[10] Costa E, Swinkels DW, Laarakkers CM, Rocha-Pereira P, Rocha S, Reis F, Teixeira F, Miranda V, do Sameiro Faria M, Loureiro A, Quintanilha A (2009). Hepcidin serum levels and resistance to recombinant human erythropoietin therapy in haemodialysis patients. Acta haematologica.

[11] Papanikolaou G, Tzilianos M, Christakis JI, Bogdanos D, Tsimirika K, MacFarlane J, Goldberg YP, Sakellaropoulos N, Ganz T, Nemeth E (2005). Hepcidin in iron overload disorders. Blood.

[12] Ryu J, Eung-Jun Kim S (2012). Interleukin-6 -634 C/G and -174 G/C Polymorphisms in Korean Patients Undergoing Hemodialysis. Korean J Intern Med.

[13] Talaat RM, Abdelkhalek MS, El-Maadawy EA, Abdel-Mageed WS, El-Shenawy SZ, Osman MA (2017). Association of TNF-Alpha gene polymorphisms and susceptibility to hepatitis B virus infection in Egyptians. Human immunology.

[14] Hunter CA, Jones SA (2015). IL-6 as a keystone cytokine in health and disease. Nature immunology.

[15] Talaat RM, Abdel-Aziz AM, El-Maadawy EA, Abdel-Bary N (2015). CD38 and interleukin 6 gene polymorphism in Egyptians with diffuse large B-cell lymphoma (DLBCL). Immunological investigations.

[16] Radovic M1, Jelkmann W, Djukanovic L, Ostric V (1999). Serum erythropoietin and interleukin-6 levels in hemodialysis patients with hepatitis virus infection. J Interferon Cytokine Res.

[17] Buraczynska M, Jozwiak L, Ksiazek P, Borowicz E (2007). Interleukin-6 gene polymorphism and faster progression to end-stage renal failure in chronic glomerulonephritis. Transl. Res..

[18] Ryu JH, Kim SJ (2012). Interleukin-6 -634 C/G and -174 G/C polymorphisms in Korean patients undergoing hemodialysis. Korean J. Intern. Med..

[19] Barrett S, Collins M, Kenny C, Ryan E, Keane CO, Crowe J (2003). Polymorphisms in tumour necrosis factor-a, transforming growth factor-ß, interleukin-10, interleukin-6, interferon-, and outcome of hepatitis C virus infection. Journal of medical virology.

[20] Saifan C, El-Charabaty E, Kleiner M, El-Sayegh S (2013). Effect of hepatitis C virus infection on erythropoiesis in patients on hemodialysis. International journal of nephrology and renovascular disease.

[21] Fujita N, Sugimoto R, Motonishi S, Tomosugi N, Tanaka H, Takeo M, Iwasa M, Kobayashi Y, Hayashi H, Kaito M, Takei Y (2008). Patients with chronic hepatitis C achieving a sustained virological response to peginterferon and ribavirin therapy recover from impaired hepcidin secretion. Journal of hepatology.

[22] Ibrahim M, Gadalla H, Raslan H, Abou Ellel H, William E (2009). Effect of hepatitis-C virus infection on serum hepcidin in hemodialysis patients.

[23] Shan Y, Lambrecht RW, Bonkovsky HL (2005). Association of hepatitis C virus infection with serum iron status: analysis of data from the third National Health and Nutrition Examination Survey. Clinical infectious diseases.

[24] Sabry A, El-Dahshan K, Mahmoud K, El-Husseini A, Sheashaa H, Abo-Zenah H (2007). Effect of hepatitis C virus infection on haematocrit and haemoglobin levels in Egyptian hemodialysis patients. Eur J Gen Med.

[25] Bennermo M, Held C, Green F, Strandberg LE, Ericsson CG, Hansson LO, Watkins H, Hamsten A, Tornvall P (2004). Prognostic value of plasma interleukin-6 concentrations and the- 174 G&gt; C and- 572 G&gt; C promoter polymorphisms of the interleukin-6 gene in patients with acute myocardial infarction treated with thrombolysis. Atherosclerosis.

[26] Nasr MY, Deeb AS, Badra G, El Sayed IH (2016). Lack of Any Relationship Between Circulating Autoantibodies and Interleukin-6 Levels in Egyptian Patients Infected with the Hepatitis C Virus. Asian Pacific journal of cancer prevention: APJCP.

[27] Giannitrapani L, Soresi M, Giacalone A, Campagna ME, Marasa M, Cervello M, Marasa S, Montalto G (2011). IL-6- 174G/C polymorphism and IL-6 serum levels in patients with liver cirrhosis and hepatocellular carcinoma. Omics: a journal of integrative biology.

[28] Giannitrapani L, Soresi M, Balasus D, Licata A, Montalto G (2013). Genetic association of interleukin-6 polymorphism (-174 G/C) with chronic liver diseases and hepatocellular carcinoma. World journal of gastroenterology: WJG.

[29] Morozumi T, Sharma A, De Nardin E (2009). The Functional Effects of the- 455G/A Polymorphism on the IL-6-Induced Expression of the ß-fibrinogen Gene may be due to Linkage Disequilibrium with Other Functional Polymorphisms. Immunological investigations.

[30] Terry CF, Loukaci V, Green FR (2000). Cooperative influence of genetic polymorphisms on interleukin 6 transcriptional regulation. Journal of Biological Chemistry.

[31] Hirschhorn JN, Lohmueller K, Byrne E, Hirschhorn K (2002). A comprehensive review of genetic association studies. Genetics in Medicine.

[32] Rothman N, Skibola CF, Wang SS, Morgan G, Lan Q, Smith MT, Spinelli JJ, Willett E, De Sanjose S, Cocco P, Berndt SI (2006). Genetic variation in TNF and IL10 and risk of non-Hodgkin lymphoma: a report from the InterLymph Consortium. The lancet oncology.

[33] Liu S, Qiu XQ, Zeng XY, Bai H, Bei CH, Yang Y (2012). Relationship between IL6-572G/C polymorphism and hepatocellular carcinoma in men. Zhonghua gan zang bing za zhi= Zhonghua ganzangbing zazhi= Chinese journal of hepatology.

[34] Park BL, Lee HS, Kim YJ, Kim JY, Jung JH, Kim LH, Shin HD (2003). Association between interleukin 6 promoter variants and chronic hepatitis B progression. Experimental & molecular medicine.

[35] Falleti E, Fabris C, Vandelli C, Colletta C, Cussigh A, Smirne C, Fontanini E, Cmet S, Minisini R, Bitetto D, Toniutto P (2010). Genetic polymorphisms of interleukin-6 modulate fibrosis progression in mild chronic hepatitis C. Human immunology.

[36] Cussigh A, Falleti E, Fabris C, Bitetto D, Cmet S, Fontanini E, Bignulin S, Fornasiere E, Fumolo E, Minisini R, Pirisi M (2011). Interleukin 6 promoter polymorphisms influence the outcome of chronic hepatitis C. Immunogenetics.

[37] Lu Y, Wu Z, Peng Q, Ma L, Zhang X, Zhao J, Qin X, Li S (2014). Role of IL-4 gene polymorphisms in HBV-related hepatocellular carcinoma in a Chinese population. PloS one.

[38] Zhang X, Rovin BH (2010). Hepcidin expression by human monocytes in response to adhesion and pro-inflammatory cytokines. Biochimica et Biophysica Acta (BBA)-General Subjects.

[39] Girndt M1, Stenvinkel P, Ulrich C, Axelsson J, Nordfors L, Barany P, Carrero JJ, Heine GH, Kaul H, Köhler H (2007). Influence of cytokine gene polymorphisms on erythropoetin dose requirements in chronic haemodialysis patients. Nephrol Dial Transplant.

[40] Balakrishnan VS, Guo D, Rao M, Jaber BL, Tighiouart H, Freeman RL, Huang C, King AJ, Pereira BJ, HEMO Study Group (2004). Cytokine gene polymorphisms in hemodialysis patients: association with comorbidity, functionality, and serum albumin. Kidney international.

